# Calcium Alginate Beads with Entrapped Iron Oxide Magnetic Nanoparticles Functionalized with Methionine—A Versatile Adsorbent for Arsenic Removal

**DOI:** 10.3390/nano11051345

**Published:** 2021-05-20

**Authors:** Surbhi Lilhare, Sunitha B. Mathew, Ajaya K. Singh, Sónia A. C. Carabineiro

**Affiliations:** 1 Department of Chemistry, Govt. V. Y. T. PG Autonomous College, Durg, Chhattisgarh 491001, India; surbhililhare987@gmail.com; 2 LAQV-REQUIMTE, Department of Chemistry, NOVA School of Science and Technology, Universidade NOVA de Lisboa, 2829-516 Caparica, Portugal; sonia.carabineiro@fct.unl.pt

**Keywords:** arsenic (III), adsorption, magnetic nanoparticles, methionine functionalized, calcium alginate, spectrophotometric method

## Abstract

A novel beads adsorbent, consisting of calcium alginate entrapped on magnetic nanoparticles functionalized with methionine (MFMNABs), was developed for effective elimination of arsenic from water. The material was characterized by FT-IR (Fourier Transform Infrared Spectroscopy), SEM (Scanning Electron Microscopic), XRD (X-ray Diffraction) and TEM (Transmission Electron Microscopy). The arsenic removal capacity of the material was studied by altering variables such as pH of the solution, contact time, adsorbent dose and adsorbate concentration. The maximal removal of As(III) was 99.56% under optimal conditions with an equilibrium time of 110 min and pH 7.0–7.5. The adsorption followed a second order kinetics and data best fitted the Langmuir isotherm with a correlation coefficient of R^2^ = 0.9890 and adsorption capacity (q_m_) of 6.6533 mg/g. The thermodynamic study showed entropy change (∆S) and enthalpy change (∆H) to be 34.32 J mol^−1^ K and 5.25 kJ mol^−1^, respectively. This study proved that it was feasible to treat an As(III) solution with MFMNABs. The synthesized adsorbent was cost-effective, environmentally friendly and versatile, compared to other adsorbents. The adsorption study was carried by low cost spectrophotometric method using N- bromosuccinimide and rhodamine-B developed in our laboratory.

## 1. Introduction

Arsenic is naturally present in the crust of Earth and has long been recognized as highly toxic and carcinogenic, affecting millions of humans in the world [1,2]. Long-term exposure to arsenic gives symptoms such as vomiting, abdominal pain, diarrhea, severe gastrointestinal irritation, gastrointestinal damage, cardiac damage and several types of cancer [3]. It can result in vascular diseases like black foot disease [4,5]. Arsenic can be found in both organic and inorganic forms, as trivalent arsenite (H_3_AsO_3_, HAsO_3_^2−^ or H_2_AsO_3_^−^) and pentavalent arsenate (H_3_AsO_4_, HAsO_4_^2−^, H_2_AsO_4_^−^ or AsO_4_^3−^), of which the first form, As(III) is more noxious than As(V) [6]. In oxidizing conditions, arsenite converts to arsenate, and vice versa under reducing conditions. Inorganic arsenic compounds are more harmful and toxic, compared to organic arsenic compounds [7]. Inorganic and organic arsenic compounds are mainly used to preserve wood and as pesticides [8,9]. It also finds applications in many industries such as pharmaceuticals, paints, pesticide production, leather, textiles, etc. Several food supplements and care products contain trace amounts of arsenic and it is also used in medical products [10]. The maximum permissible limit for As in drinking water is 10 µg/L, as defined by the World Health Organization [11].

Several techniques were reported for elimination of arsenic from water, namely oxidation [12], alum and iron coagulation [13,14], adsorption and ion-exchange (iron-coated sand, activated alumina and ion-exchange resin) [15,16], filtration with membranes [17] and reverse osmosis [17]. Most of the methods suffer several shortcomings. Among these technologies, adsorption is cost effective, easy to operate, highly efficient and most popular, since a variety of adsorbents are available [18,19]. Several materials were reported for As(III) removal, like pyrite fines, activated alumina, fly ash, manganese greensand [20], meso porous silicas with amino-functionalizations [21], Al-loaded Shirasu-zeolite [22], clinoptilolite and other zeolites [23,24,25]. However, they suffer from some disadvantages, like complexity, high cost, etc.

Alginate has several advantages as it is cheap and easily forms cross-linking with a CaCl_2_ solution. Ca^2+^ cations are able to bind two carboxyl moieties of guluronic remains in alginate chains. It is a natural product (natural polysaccharide extracted from brown seaweeds), which is non‒toxic, inexpensive, biodegradable, biocompatible and water soluble. It has been largely used for immobilization of activated carbon (C) [26], carbon nanotubes (C-NT) [27], nanoparticles of TiO_2_ [28] and magnetite [29], generating novel adsorbents to eliminate heavy metals, pigments and dyes from wastewater. Pure Magnetic Nanoparticles (MNPs) are not applied directly on account of the robust dipole–dipole attractions between the MNPs and the large surface area that might lead to aggregation during the adsorption process. Therefore, magnetic nanoparticles are entrapped into several types of stabilizers (organic and inorganic), such as activated carbon [30], chitosan [31,32,33,34], β-cyclodextrin [35,36] and alginate biopolymer [37,38,39]. In this work, methionine, a sulfur containing amino acid, has been incorporated along with alginate.

In the present study, a new adsorbent MFMNABs (calcium alginate beads with entrapped iron oxide magnetic nanoparticles functionalized with methionine) has been synthesized and applied for the removal of As(III). MFMNABs was found to be a cheap, ecofriendly adsorbent for the elimination of arsenic (III) with comparable adsorption capacity and recoverability. It is effective given its large surface area and occurrence of surface amino groups. This adsorbent is evaluated with respect to various variables like temperature, time, pH, etc. in batch conditions. The adsorption isotherms, kinetics and thermodynamic studies have been performed and found to have advantages like cheapness, simplicity, good reproducibility and high adsorption. Adsorption studies of As(III) using ultraviolet-visible (UV-Vis) spectrophotometry and atomic absorption spectroscopy are reported [40,41]. Herein, a simple spectrophotometric method developed in our laboratory using N-bromosuccinimide and rhodamine-B is used for investigating the adsorption process.

## 2. Materials and Methods

### 2.1. Materials and Reagents

Sodium arsenite (NaAsO_2_) (Merck, Mumbai, India), methionine (C_5_H_11_NO_2_S) (Merck, Mumbai, India), N-bromosuccinimide (NBS) (Schmid and Co., Freudenstadt, West Germany), rhodamine-B (S.D. Fine Chem. Ltd., Mumbai, India), sodium alginate (C_6_H_9_NaO_7_) [CDH, Delhi, India], ferric chloride hexahydrate (FeCl_3_·6H_2_O) [CDH, Delhi, India], ferrous chloride dihydrate (FeCl_2_·2H_2_O) [CDH, Delhi, India], NH_4_OH [AR grade, Mumbai, India] and HCl (Loba Chemie, Mumbai, India) were used. The chemicals were all analytical grade and no further purification was made before use. Double distilled water (DDW) was utilized.

A stock solution of As(III) (1000 mg L^−1^) was obtained by dissolution of 0.1732 g of analytical grade NaAsO_2_ in 100 mL of DDW. A 0.002 M N-bromosuccinimide solution was obtained by dissolution of 0.178 g of NBS in 100 mL distilled water and kept in an amber colored bottle. The required working solutions of NBS were prepared by dilution of the stock solution. A 0.001 M of rhodamine-B solution was prepared. Hydrochloric acid was diluted with DDW to get 0.01 M HCl.

### 2.2. Equipments

X-ray diffraction (XRD) (Expert-Pro PW3064/60, Raipur, India) analysis of powdered samples was done at 30°–80° and PANalytical 3 kW X’pert Powder-Multifunctional. Fourier transform-infrared spectrometer (FT-IR) (Thermo Nicolet Avtar 370, Cochin, India) was used to obtain the infrared spectra in the 400–4000 cm^−1^ range using KBr pellets. Scanning Electron Microscopy (SEM) images of MFMNABs were obtained before and after adsorption (Jeol 6390LA/OXFORD XMX N, Cochin, India). The details of shape and characteristics features was obtained from Transmission Electron Microscopy (TEM) images (Jeol/JEM 2100, Cochin, India). Systronic UV-visible spectrophotometer-117 (Carry 50 scan, Varian, Durg, India) with 1 cm quartz cell (0.1 mL) was used for the measurement of absorbance. The pH was measured by a digital pH meter (Systronics model-112, Durg, India).

### 2.3. Synthesis of Methionine Functionalized Magnetic Nanoparticles (MFMNPs)

The nanoparticles (NPs) were obtained by co-precipitation [42]. Fe(II) and Fe(III) were co-precipitated by ammonia in hydrothermal conditions. A total of 2.4 g of ferrous chloride and 4.8 g of ferric chloride were separately dissolved in 50 mL DDW and 15 mL of 1.5 M NH_4_OH was added dropwise at 25–30 °C with stirring, at pH 10. The black precipitate obtained was separated magnetically and washed 4–5 times with DDW. To this 10 mL of 0.1% methionine in double distilled water was added dropwise. The content was heated up to 80 °C over 30 min with stirring. The obtained methionine functionalized magnetic nanoparticles were separated by applying an external magnet, properly washed with DDW and dried for 2 h at 250 °C.

### 2.4. Preparation of Calcium Alginate Beads with Entrapped Iron Oxide Magnetic Nanoparticles Functionalized with Methionine (MFMNABs)

**Figure 1:**
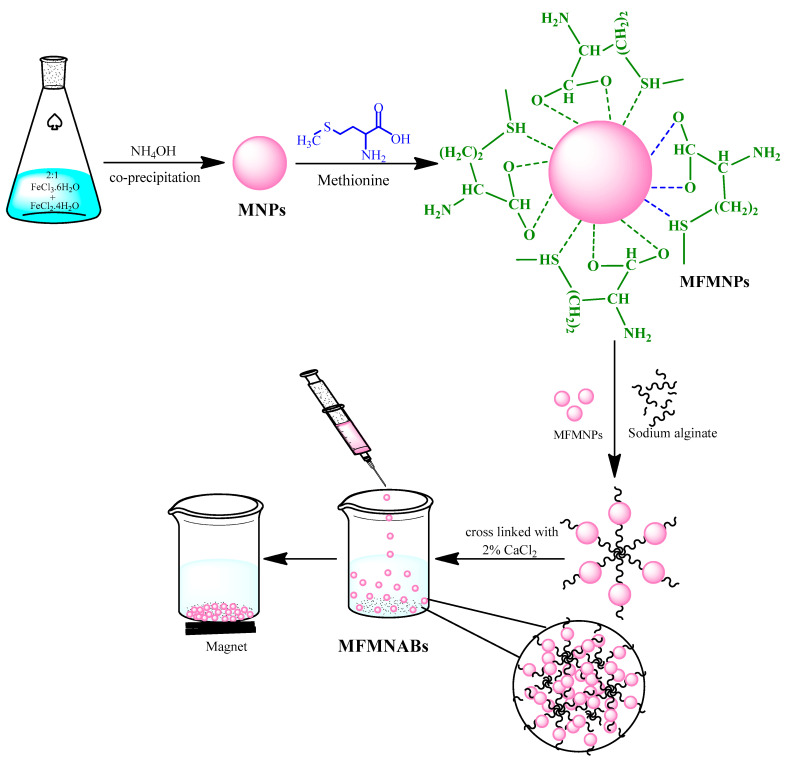
Synthetic pathway of MFMNABs.

A total of 1.5 g sodium alginate was dissolved in 50 mL DDW with stirring for 2 h to yield a viscous homogenous solution. Then, 1 g of methionine modified Fe_3_O_4_ NPs was added with stirring. Thereafter the mixture was added dropwise to the CaCl_2_ solution, and MFMNABs were obtained. To get stable beads, the gel beads were kept in CaCl_2_ solution for 24 h. The beads were washed several times with DDW and stored in DDW for later use. The color of beads was reddish brown given the entrapping of the magnetic NPs modified by alginate. The synthesis of MFMNABs is presented in Figure 1.

### 2.5. Procedure for As(III) Analysis

After adsorption, the beads and liquid were separated by normal filter paper and the amount of As(III) in solution was determined spectrophotometrically. To 5 mL of the filtrate 0.002 M NBS solution (2.5 mL) was added, where NBS oxidizes As(III) and the unconsumed NBS, corresponding to the concentration of As(III) was determined by addition of 0.001 M of rhodamine-B (4 mL). The mixture was left for 5 min. The unconsumed NBS bleached the color of rhodamine-B and absorbance was measured at 555 nm.

### 2.6. Batch Adsorption Studies

Batch adsorption studies indicate that As(III) can be adsorbed onto the prepared modified alginate beads. The experimental parameters were optimized by investigating the various parameters like solution pH (4.0–9.0), time of contact (15–120 min), dosage of adsorbent (0.1–2.0 g) and concentration of As(III) in solution (10–35 mg/L). The pH was adjusted by 0.1 N HCl and 0.1 N NaOH. The appropriate amount of adsorbent (MFMNABs) was added to an aliquot containing a known amount of As(III) with the intended initial pH and adequate contact time to reach equilibrium. The absorbance was measured by UV-Vis spectrophotometry at 555 nm. The % amount of As(III) removal was obtained by Equation (1):

(1) $$\%\;\mathrm{Removal}=\frac{C_{0}-C_{e}}{C_{0}}\times 100$$

While the quantity of adsorbed As(III) (*q_e_*) was determined from Equation (2):

(2) $$q_{e}=\frac{(C_{0}-C_{e})\;V}{m}$$

where *C*_0_ is the initial concentration and *C_e_* is the equilibrium concentration of As(III) (μg/mL), m being the mass of adsorbent (g) and *V* the solution volume (L) [43].

### 2.7. Determination of pHpzc (Point of Zero Charge)

In a series of 50 mL conical flasks containing 10 mL of 0.01 M NaCl, 0.1 N of NaOH solution was added to adjust the initial pH (pH_i_) in the range 4–9. Then, to each flask, 1.6 g MFMNABs was added and shaken for 24 h on a rotatory shaker at 150 rpm and the final pH (pH_f_) was noted. The difference between the initial and final pH (ΔpH = pH_i_ − pH_f_) was plotted against the initial pH (pH_i_) of the solution. The pH on the horizontal line in the plot corresponding to ΔpH equal to zero gives pHpzc.

### 2.8. Adsorption Isotherm

The adsorption efficiency was determined from adsorption isotherms. The adsorption is studied by obtaining the equilibrium concentration using various isotherms [44,45]. Herein, the most common isotherms, namely, Frendlich [46], Langmuir [47] and Temkin [48] were used to find the best model fitting.

Langmuir isotherm: This model was used to assess the adsorption process using Equation (3):

(3) $$\frac{1}{q_{e}}=\frac{1}{q_{m}}+\frac{1}{K_{m}\cdot q_{e}}\cdot \frac{1}{C_{e}}$$

where *K_m_*—Langmuir adsorption constant (L/mg) and *q_m_*—maximum adsorption capacity of the adsorbent (mg/g). *C_e_* and *q_e_*—equilibrium concentration and equilibrium adsorption capacities (mg/g) of As(III) ions, respectively. The value of *R*^2^ was obtained from the plot 1/*q_e_* vs. 1/*C_e_*. In addition to this, the dimensional separation factor (*R_L_*) was calculated to characterize isotherms using Equation (4):

(4) $$R_{L}=\frac{1}{1-K_{L}C_{0}}$$

where *C*_0_ refers to the initial concentration of adsorbate and *K_L_* is the rate of adsorption. The *R_L_* value infers that adsorption was irreversible (*R_L_* = 0), favorable (0 < *R_L_* < 1) linear, (*R_L_* = 1) or unfavorable (*R_L_* > 1). [49,50,51].

Freundlich isotherm: This model was used for investigating the adsorption capacity on heterogeneous surfaces and formation of monolayer. It is expressed by Equation (5):

(5) $$\log q_{e}=\log K_{F}+\frac{1}{n}\log C_{e}$$

where *K_F_* (L/mg) and *n* are Freundlich constants signifying, respectively, the adsorption capacity and intensity of the system. *C_e_* and *q_e_* are equilibrium concentration and equilibrium adsorption capacity (mg/g) of As(III) ions, respectively. *K_F_* and 1/*n* are calculated from the slope and intercept of log *q_e_* versus log *C_e_* plot, respectively.

Temkin isotherm: This model is based on the surface coverage and expressed by Equation (6):

(6) $$q_{e}=B_{1}\ln K_{T}+B_{1}\ln C_{e}$$

where *B*_1_ = *RT*/*b*, *B*_1_ is the Temkin constant dealing with the heat of adsorption (kJ/mol), *T* represents absolute temperature (*K*), *R* the gas constant (8.314 J/mol K) and *K_T_* the equilibrium binding constant (L/g). The plot of *q_e_* versus ln *C_e_*, enables to determine *K_T_* and *B*_1_.

### 2.9. Adsorption Kinetics

Most of the adsorption/desorption processes of many solid substances depend on time.

Pseudo-first-order kinetic model: This model of Lagergren’s kinetic equation for the adsorption of adsorbates from liquid solutions is expressed as follows [52]:

(7) $$\log\left(q_{e}-q_{t}\right)=\log q_{e}-\frac{k_{1}t}{2.303}$$

where *q_e_* (mg/g) and *q_t_* (mg/g) are quantities adsorbed at equilibrium and time *t* (min), respectively, and *k*_1_ (min^−1^) is the rate constant for pseudo-first-order equation. The values of *k*_1_ and *q_e_* were calculated by plotting log (*q_e_* _−_ *q_t_*) versus time (t).

Pseudo-second-order kinetic model: This model assumes that adsorption is controlled by chemical adsorption [53]:

(8) $$\frac{t}{q_{t}}=\frac{t}{k_{2}q_{e}^{2}}-\frac{1}{q_{e}}$$

where *q_e_* and *q_t_* are adsorption capacity (mg/g) at equilibrium and time *t* (time), and *k*_2_ (g/g per min) is the rate constant. The values of *k*_2_ and *q_e_* are calculated from the slope and intercept of the *t*/*q_t_* versus *t* plot.

Intraparticle diffusion kinetic model: The intraparticle (pore) diffusion mechanism of As(III) was studied by using the Weber and Morris model of diffusion [54], following the equation given by:

(9) $$q_{t}=K_{d}t^{1/2}+C$$

where *K_d_* (mg/g min^1/2^) is the diffusion rate constant; *C* (mg/g) is the intercept in the diffusion model. The slope and intercept were obtained by plotting *q_t_* versus √*t*.

Elovich kinetic model: This model applies satisfactorily to the chemisorption process, which implies multilayer adsorption [55] and is expressed by Equation (10).

(10) $$q_{t}=\alpha +\beta \ln t$$

where *q_t_* (mg/g) is the amount of As(III) adsorbed for time *t* (min), *α* (mg/g min^−1^) and *β* (g/mg) are obtained from the slope (*β*) and intercept (*α*) of the linear plot of *q_t_* versus ln *t*.

### 2.10. Regeneration Studies

Desorption studies were made to assess the regeneration capacity of the adsorbent. MFMNABs (1.6 g) were placed in a 100 mL conical flask with 10 µg/mL concentration of As(III) and shaken for 110 min in an incubator shaker at 30 °C. Beads were separated by a magnet and the concentration of arsenic was measured. Then, MFMNABs were recycled by washing with 0.1 N NaOH and then three times with DDW. The beads were then again added to a fresh As(III) solution and the reusability was verified.

## 3. Results and Discussion

### 3.1. Adsorbent Characterization

#### 3.1.1. X-ray Diffraction

**Figure 2:**
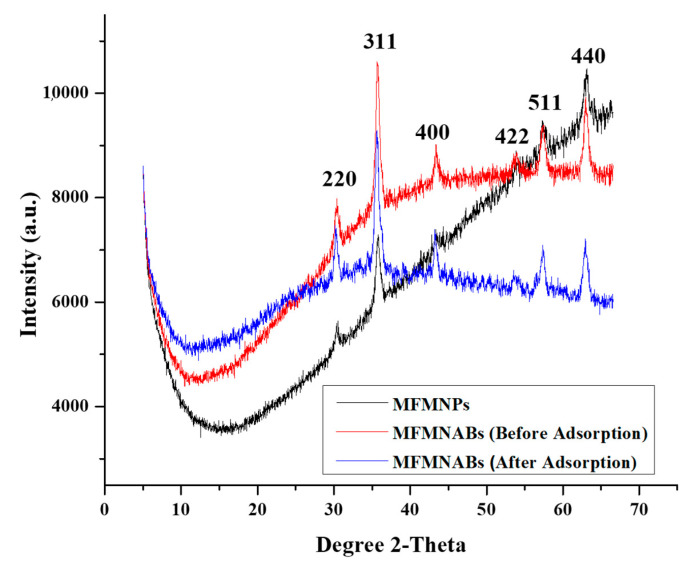
XRD diffractograms of MFMNPs and MFMNABs (before and after adsorption).

X-ray diffraction (XRD) patterns of MFMNPs, MFMNABs (before adsorption) and MFMNABs (after adsorption) show characteristic peaks, as depicted in Figure 2. The XRD diffraction pattern obtained for MFMNPs and MFMNABs (before and after adsorption) exhibits consistent peaks at (220), (311), (400), (422), (511) and (440) which is identical to that Standard JCPDS data [56] reported for Fe_3_O_4_ nanoparticles as well as the methionine-coated Fe_3_O_4_ nanoparticles [57,58]. The materials show sharp peaks, indicating that particles are crystalline and of small size and these results matched with the planes of the cubic structure of Fe_3_O_4_ (face-centered cubic) [57].

The average crystal size (D) of MFMNPs, MFMNABs (before adsorption) and MFMNABs (after adsorption) were determined by the Debye-Scherrer’s Equation (11) [59]:

(11) $$D=\frac{K\cdot \lambda }{\beta \cdot cos\theta }$$

where *D* is average crystal size in Å, *θ* is the peak angle, *β* is FWHM (Full Width at Half Maximum) of the sharp peaks, *λ* is the wavelength of X-rays (1.54 Å) and *K* is constant (equal to 0.9). The results are presented in Table 1. The XRD results show that the average size of the particles of MFMNPs was 17.04 nm and after crosslinking with alginate, the mean size of the MFMNABs particles was reduced to 12.95 nm. After adsorption, the average size of MFMNABs was 20.68 nm. The d-spacing and particle size obtained from HR-TEM and XRD data (311) are presented in Table 2.

**Figure 3:**
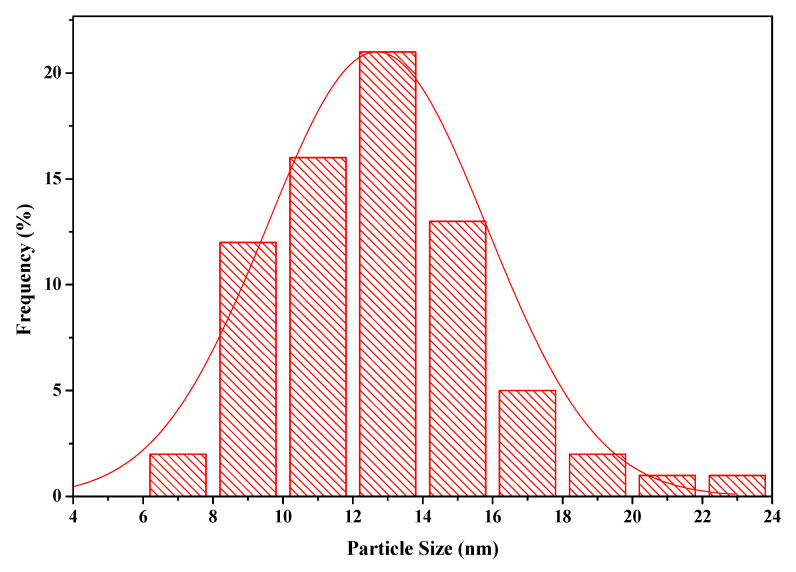
Size distribution curve of MFMNABs.

Figure 3 shows the size distribution curve of MFMNABs. It was observed that the average size calculated by the Debye–Scherrer formula (Equation (11) using XRD data (12.68 nm) is in close proximity with that calculated by the Bragg’s Equation (12) using TEM data (12.95 nm) [60].

(12) nλ=2d·sinθ

where *n* is an integer and *d* is interplanar distance.

#### 3.1.2. Fourier Transform Infrared Spectroscopy

**Figure 4:**
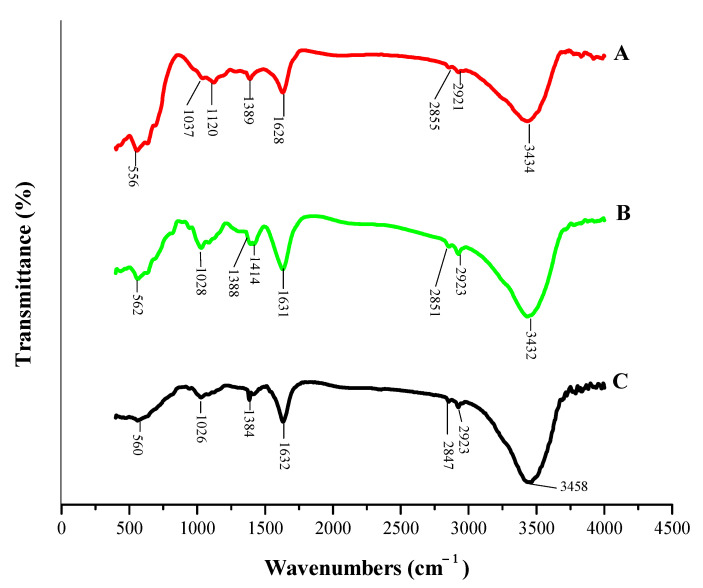
The FT-IR spectra of (**A**) MFMNPs, (**B**) MFMNABs-before adsorption and (**C**) MFMNABs-after adsorption.

MFMNPs and MFMNABs (before and after adsorption) were studied by Fourier Transform Infrared Spectroscopy (FTIR) and results are shown in Figure 4. The broad and strong peaks at 3434 cm^−1^ (MFMNPs) shifted to 3432 cm^−1^ (MFMNABs-before adsorption) and 3458 cm^−1^ (MFMNABs-after adsorption) are ascribed to stretching vibrations of ‒OH and ‒NH_2_ [61]. The weak peaks at 2921 and 2855 cm^−1^ in MFMNPs, 2923 and 2851 cm^−1^ in MFMNABs (before adsorption), and 2923 and 2847 cm^−1^ in MFMNABs (after adsorption) are ascribed to C–H stretching vibrations [62]. The peak 1628 (MFMNPs) cm^−1^ assigned to asymmetric stretching of NH_3_^+^ get shifted to 1631 cm^−1^ (MFMNABs-before adsorption) and 1632 cm^−1^ (MFMNABs-after adsorption). The additional peak at 1414 cm^−1^ in MFMNABs (before adsorption) is assigned to the symmetric stretching vibration of COO^−^ of sodium alginate [63]. The weak bands observed at 1389 cm^−1^ (MFMNPs), 1388 (MFMNABs-before adsorption) and 1384 (MFMNABs-before adsorption) were attributed to the stretching vibration of the C = O [64] bond and the band at 1120 cm^−1^ to the bending vibration of NH_3_ [65]. The peaks at 1037 cm^−1^ (MFMNPs), shifted to 1028 cm^−1^ (MFMNABs-before adsorption) and 1026 cm^−1^ (MFMNABs-after adsorption) indicate C–O stretching vibrations [66]. The presence of a band at 556 cm^−1^ (MFMNPs), 562 cm^−1^ MFMNABs (before adsorption) and 560 cm^−1^ MFMNABs (after adsorption) is due to the vibration of Fe–O bond in Fe_3_O_4_ and C–S–C stretching mode [67,68].

#### 3.1.3. Scanning Electron Microscopy

**Figure 5:**
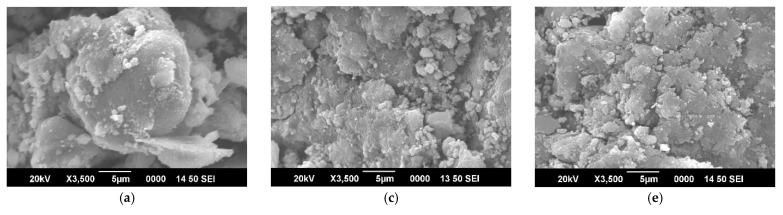

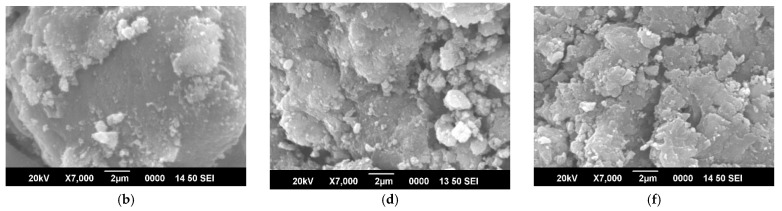
The SEM images of (**a**,**b**) MFMNPs, (**c**,**d**) MFMNABs (before adsorption) and (**e**,**f**) MFMNABs (after adsorption) under different magnification.

Scanning Electron Microscopy (SEM) was used to characterize the unmodified and modified beads (before and after adsorption). SEM images of MFMNPs and MFMNABs (before and after adsorption) taken under different magnification is shown in Figure 5. Figure 5a,b reveals that the surface of MFMNPs are much smother as compared to the surface of MFMNABs. Figure 5c,d indicate that after cross-linking with alginate, MFMNABs exhibit rough, multi-layered surface with wide cavities and irregular pores. Figure 5e,f shows that the surface of MFMNABs after adsorption become aggregated with narrow cavities.

#### 3.1.4. Transmission Electron Microscopy

**Figure 6:**
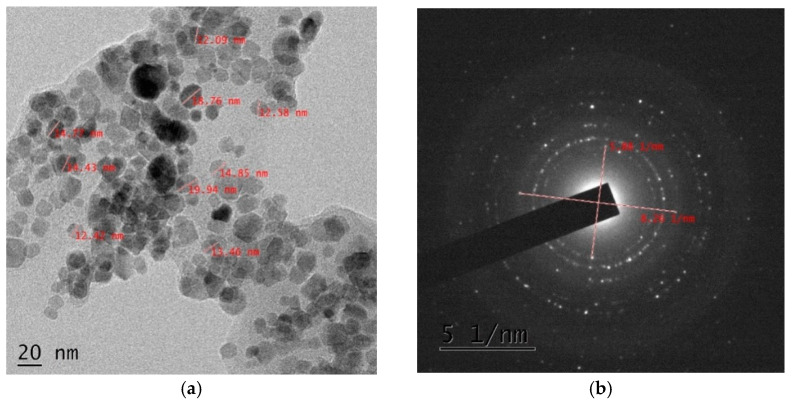
Transmission Electron Micrograph (TEM) (**a**) and SAED patterns (**b**) of MFMNABs.

The shape and size of adsorbent were examined by transmission electron microscopy (TEM). A matrix with spherical or ellipsoidal particles smaller than 20 nm is seen (Figure 6). The distribution curve shows that the sizes of particles are distributed in the range of 6–20 nm and the majority of particle sizes are between 12–14 nm (Figure 3). Most particles were scattered but some are aggregated indicating stabilization. The structure of the synthesized functionalized beads was examined with selected area electron diffraction (SAED). The contrast image show diffraction rings with bright spots depicting the polycrystalline nature of the adsorbent.

### 3.2. pH Effect

**Figure 7:**
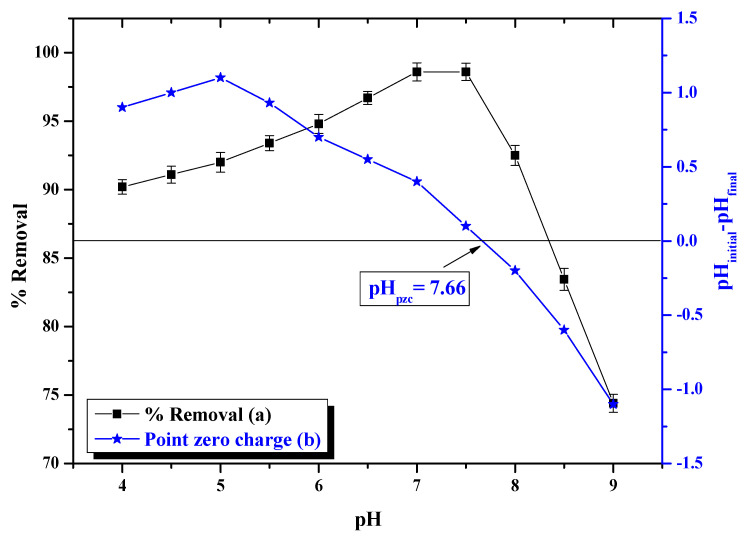
(**a**) pH effect on the adsorption of As(III) by MFMNABs at initial concentration of 10 µg/mL, adsorbent dose; 1.0 g, contact time; 110 min and, (**b**) pH_pzc_ (point zero charge) of MFMNABs.

pH is important for adsorption, as it affects the adsorption capacity of the adsorbent. The effect of pH on As(III) removal efficiency is shown in Figure 7a. Removal of As(III) was investigated by varying the pH ranging from 4 to 9 under the following conditions: 10 mg/L initial As(III) concentration, 1.0 g dosage, 110 min contact time and room temperature (~35 °C). The pH of the sample was adjusted using 0.1 N NaOH or 0.1 N HCl. The percentage removal was obtained at equilibrium and it was found that the percentage removal (%) was maximal at pH 7.0–7.5. Thus, pH 7 was used for further studies.

In an aqueous solution, the As(III) species formed are likely to be H_3_AsO_3_ and H_2_AsO_3_^−^ or HAsO_3_^2−^ anionic forms. Below 9.2, the non-ionic H_3_AsO_3_ is the dominant species of As(III) and weak Van der Waals forces are expected between As(III) and MFMNABs. As the pH approaches 7, the amounts of anionic species H_2_AsO_3_^−^ tend to increase, resulting in more specific binding, leading to enhanced removal of As(III) [69]. The point of zero charge (pH_pzc_) for MFMNABs value was found to be 7.66 (Figure 7b). The surface is positively charged below this value and small amounts of anionic species are adsorbed due to electrostatic attraction in the pH range 7.0 to 7.5. However, when the pH is higher than the pH_pzc_ value, the adsorbent surface is negatively charged, causing the repulsion force [70,71]. Adsorption may also be due to strong chelation via sulfur and −NH_2_ group of the adsorbent. N and S atoms are potent donors due to presence of lone pair of electrons [72,73].

The enhanced removal of As(III) at pH 7.0–7.5 may also be attributed to the formation of an outer-sphere complex by hydrogen bonding or probably because of inner sphere complex formation through ligand exchange with a hydroxyl group (−OH) on the adsorbent surface. In acidic pH, the highly protonated adsorbent surface is less favourable for inner-sphere complex formation by As(III), which leads to a decrease in removal capacity [74]. At basic pH, presence of large amounts of OH^−^ may compete for adsorption sites with anionic As(III) species which affects the removal capacity of As(III) [74].

### 3.3. Adsorbent Dose Effect

**Figure 8:**
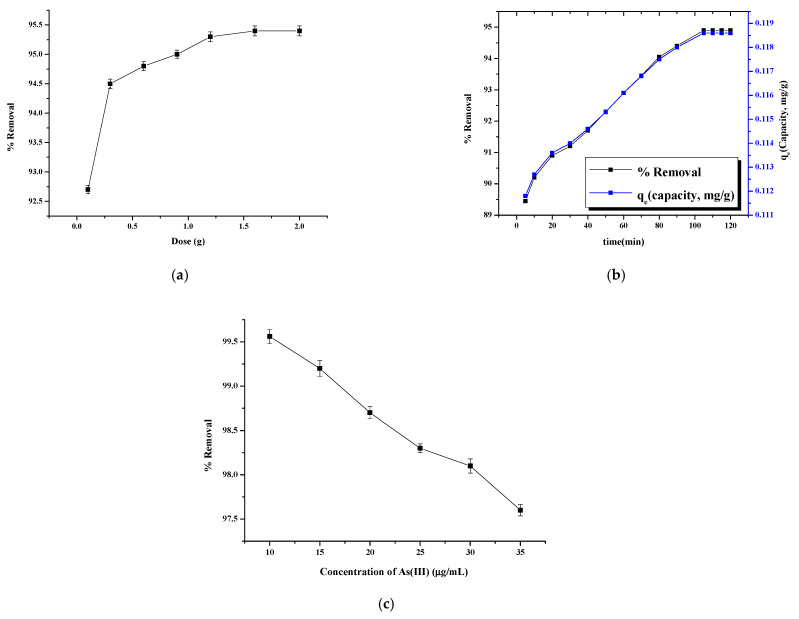
Effect of Adsorbent dose (**a**) contact time (**b**) and initial concentration (**c**) on the % removal using MFMNABs. Conditions: (**a**) 10 µg/mL As(III) concentration; pH 7, contact time 110 min, (**b**) As(III) 10 µg/mL; adsorbent dose 1.6 g; and pH 7 and (**c**) Adsorbent dose; 1.6 g, contact time 110 min and pH 7.

The adsorbent dose effect on the As removal was studied and results are shown in Figure 8a. The elimination of As(III) in water was investigated by varying the amounts of adsorbent (0.1–2.0 g). The removal efficiency improved by augmenting the adsorbent amount from 0.1 to 1.6 g. The greater the number of vacant sites on the adsorbent, the greater the capacity of adsorption. As the dosage increased from 0.1 g to 1.6 g in 10 µg/mL of As(III) ions at pH 7, the % removal increased from 92.7% to 95.4%, but afterwards, the removal remained constant. The optimal dose was 1.6 g.

### 3.4. Contact Time Effect

The removal of As(III) at diverse timings is found in Figure 8b. A total of 10 mL of As(III) solution (10 mg/L) was taken at 7 pH and 1.6 g of adsorbent was added. The extraction of As(III) increases from 90.2% to 94.9% then reaching the steady state in 105 min. After equilibrium, the amount removed remains the same though the agitation time is increased to 120 min. The availability of a large number of vacant sites initially leads to rapid adsorption. However, with time, the number of vacant sites diminish, and elimination slows. It was observed that the maximum % removal of 94.9% was obtained at 110 min.

### 3.5. Initial Concentration Effect

The adsorption of As(III) was followed by varying the arsenic amount (10–35 µg/mL), with 1.6 g dosage, 110 min contact time and room temperature (~35 °C). In general, the amount of As(III) removed by MFMNABs first augmented with the rise of the initial concentration of arsenic and the optimal value of 99.56% was observed at 10 µg/mL arsenic concentration. More active sites are available for adsorption of As(III) ions at lower concentrations. With the number of As(III) ions being increased at higher concentrations, less active sites are available for adsorption. Figure 8c shows that As(III) adsorption is dependent of its initial amount, and when this quantity increases, the adsorption is lower. With an increase in the initial amount of As(III), these ions compete for free sites of the adsorbent, which results in the saturation of more sites. There is a very slight improvement in adsorption upon an increase in the arsenic amount, more noticeable for low concentrations (5–10 µg), showing high affinity [75].

### 3.6. Adsorption Isotherm

**Figure 9:**
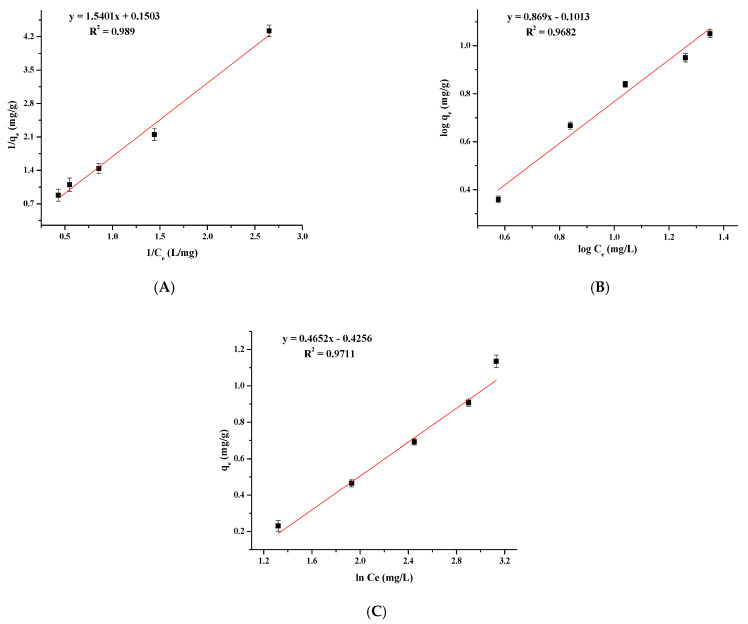
Adsorption isotherms of (**A**) Langmuir, (**B**) Freundlich and (**C**) Temkin for As(III) adsorption onto MFMNABs.

Adsorption isotherms were analyzed using the Langmuir (Figure 9A), Freundlich (Figure 9B) and Temkin (Figure 9C) isotherm models to evaluate the best fit with the correlation coefficients (R^2^). Results are presented in Table 3. The data showed that Langmuir isotherm model was the best fit with a R^2^ = 0.9890, compared to Freundlich (R^2^ = 0.9682) and Temkin (R^2^ = 0.9711) models, proving the homogeneous surface. The maximum adsorption capacity was 6.6533 mg/g for the MFMNABs. In addition, the value of the dimensional separation factor (R_L_) was found to be less than 1, which confirms that the adsorption of As(III) is a favorable process.

### 3.7. Adsorption Kinetics

**Figure 10:**
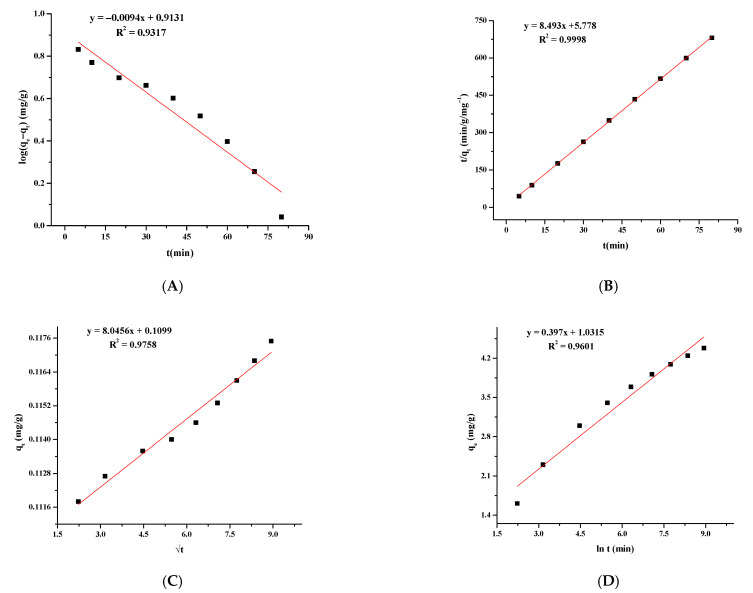
Adsorption of As(III) onto MFMNABs fitted with the pseudo-first order (**A**), pseudo-second order (**B**) intra-particle diffusion (**C**) and Elovich (**D**) models.

Adsorption of As(III) by MFMNABs was modeled using pseudo-first-order (Figure 10A), pseudo-second-order (Figure 10B), Elovich (Figure 10C) and intra-particle diffusion (Figure 10D) models to explain the kinetic data. The values of the kinetic model parameters are listed in Table 4. The pseudo second order kinetic model shows the highest R^2^ value of 0.9998. The kinetic results agree with those of different carbon and clay-based adsorbents [76,77,78].

### 3.8. Thermodynamic Parameters

**Figure 11:**
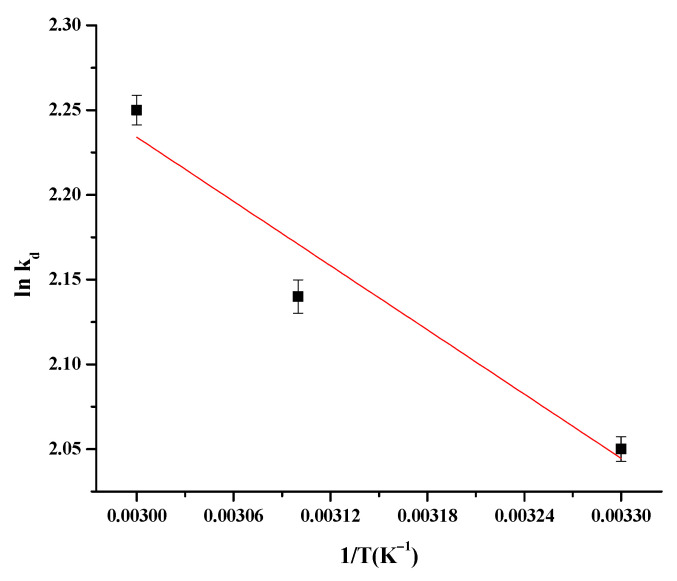
Van’t Hoff’s plot for adsorption of As(III) on MFMNABs.

A rise in temperature caused an enlargement in the rate of As(III) adsorption proving [78] that the process is endothermic [79]. The adsorption thermodynamic parameters, i.e., Gibbs free energy (ΔG), changes in enthalpy (ΔH) and changes in entropy (ΔS) were calculated by the equations:

(13) $$∆G=-\mathrm{RT}\ln K_{d}$$

(14) $$\ln K_{d}=\frac{\Delta S}{R}-\frac{\Delta H}{\mathrm{RT}}$$

where K_d_—equilibrium constant of the Langmuir model (L/g), T—absolute temperature (Kelvin, k) and R—universal gas constant (8.314 kJ/mol K). The values of ∆H and ∆S were 5.25 KJ/mol and 34.32 J/mol/K, respectively, taken from the slope and the intercept of the linear plot of ln K_d_ versus 1/T (Figure 11). ∆H has a positive value, confirming the endothermicity of the adsorption and the positive values of ∆S suggest a randomness increase. A similar endothermic adsorption behavior is found in many pollutant adsorption systems in the literature [80,81]. The change in the Gibbs free energy (ΔG) was −1.8, −1.97 and −2.17 KJ/mol for 30, 40 and 50 °C, respectively. ∆G has a negative value, meaning that the adsorption of As on the MFMNABs is feasible and spontaneous at all temperatures.

### 3.9. Reusability

**Figure 12:**
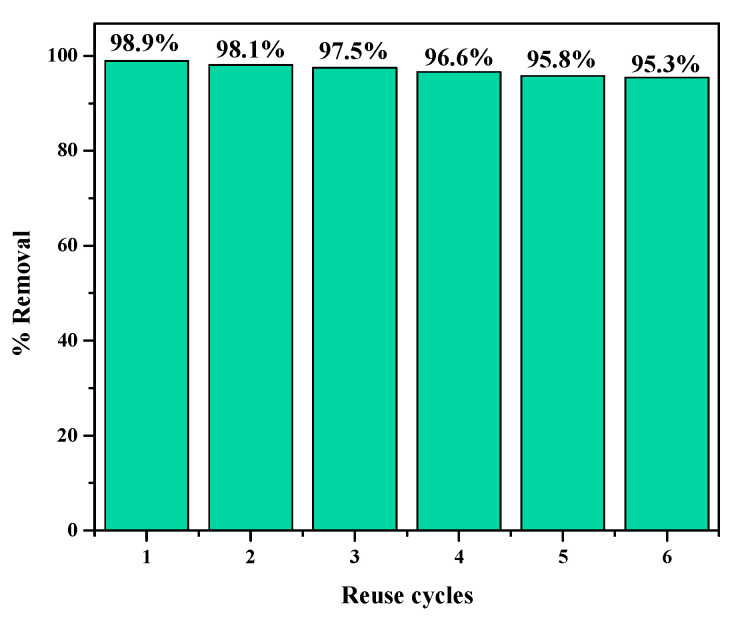
Relationship between the % removal of As(III) and reuse cycles of MFMNABs.

Desorption studies were made to assess the regeneration capacity of the adsorbent. It was found that the beads were reusable for six cycles (Figure 12).

### 3.10. Adsorption Mechanism

The FTIR spectra of MFMNABs (Figure 4) shows a shift in position and intensity of bands at 3432, 2851, 1631, 1414, 1388, 1082, 1028 and 562 cm^−1^ after adsorption of As(III). These results indicate that the O–H, NH_2_, C–H, COO^−^, C = O, C–S–C, C–O and Fe–O groups are involved in the adsorption process.

The FTIR spectra of MFMNPs exhibits bands at 1628 and 1390 cm^−1^ which is attributed to C = O and C–O stretching vibrations of the amino acid residues, respectively. The peak corresponding to C–H stretching vibrations of methionine is observed at 2870 cm^−1^. The band around 3400 cm^−1^ is assigned to the overlapping of N–H and O–H stretching peaks.

**Figure 13:**
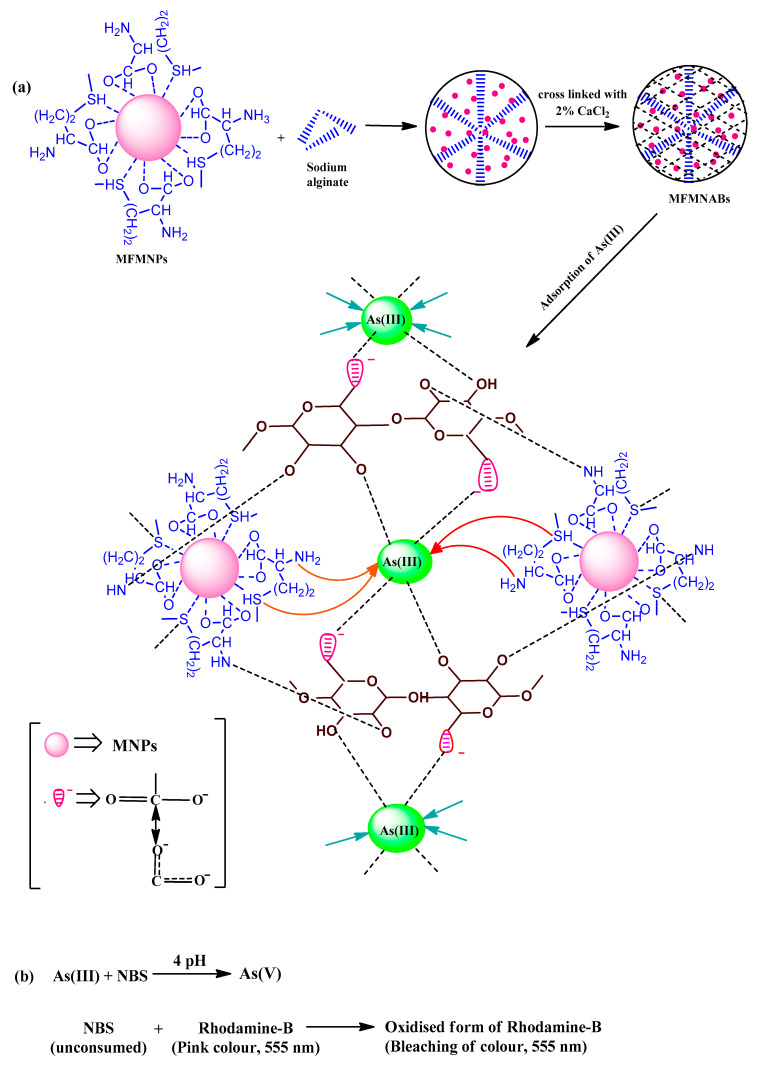
Mechanism of As(III) adsorption on MFMNABs (**a**) and reactions used to study the adsorption (**b**).

The possible interaction of methionine functionalized groups is supposed to be through N, S and O donor atoms and –OH groups, as shown in Figure 13. The carboxylate ion of amino acid can interact with Fe^3+^ by either bidentate or unidentate modalities, the former being more likely than the latter. N atom of amine group, a potent donor, can make coordinate covalent bond with Fe^3+^. Sulfur (S) atom is also expected to be a potent donor atom due to its lone pair of electrons [82].

In the acidic pH, the predominant As(III) species, H_3_AsO_3_ get adsorbed due to weak Van der Waals forces. At pH 7.0−7.5, adsorption increases due to electrostatic attraction between the positive surface and increase in anionic species. Adsorption may also be due to strong chelation via sulfur and –NH_2_ groups [72,73]. It may also be attributed to the formation of complex through hydrogen bonding. The shift in the position and decrease in intensity of Fe–O peak indicate the involvement of Fe–O bond also in the adsorption process. The scheme of mechanism of adsorption of As(III) is depicted in Figure 13a and the mechanism of spectrophotometric method used is shown in Figure 13b.

## 4. Comparisons of Adsorption Capacities (q_m_) of As(III)

The maximal adsorption capacity (q_m_) of different adsorbents for the removal of As(III) are presented in Table 5.

Many adsorbents with high adsorption capacity are reported for As (V) as well as total As whereas only few adsorbents with high adsorption capacities are reported for As (III) due to its existence as non-ionic species around 7.0 pH [91]. MFMNABs exhibited high adsorption capacity, in comparison to adsorbents, like guava leaf biomass, mango bark, FHMCA, etc. reported for As(III). A few hybrid materials like polymeric/inorganic fibrous sorbent [90] and zirconium polyacrylamide [91] show higher adsorption capacity; however, they show limitations, such as, being comparatively costlier, use fibrous ion exchangers, need tedious method of fabrication, have removal at low pH (away from general aqueous condition), etc. Hence, the adsorbent methionine functionalized magnetic nanoparticles is more adequate for the adsorption of As(III) from aqueous solutions.

## 5. Conclusions

The present work provides a cheap and environmentally friendly method for the adsorption of As(III) from aqueous samples. A spectrophotometric method based on the reaction of As(III) with N-bromosuccinimide and rhodamine-B can be used for monitoring the adsorption of As(III). The process efficiency depends on several variables, like temperature, contact time, initial adsorbent concentration and solution pH. The highest adsorption was found at pH 7.0–7.5. The maximal As removal (99.56%) was for the concentration of 10 μg/mL, room temperature (~35 °C), pH 7 and adsorbent dose 1.6 g. The obtained data for As adsorption agrees well with the Freundlich isotherm. The reported modified adsorbent shows good benefits, like good removal efficiency, high adsorption capacity, cheapness, ease of synthesis and availability as well as being promising for the elimination of arsenic from water.

## Figures and Tables

**Table 1:** Crystal size values obtained by the Debye-Scherrer’s formula.

Substance	Most Intense Peak (2θ, Degree)	Most Intense Peak (θ, Degree)	hkl	FWHM * of Most Intense Peak (β, Radian)	Size of the Particles (D, nm)
MFMNPs	35.77	17.88	311	0.0144	17.04
MFMNABs (Before adsorption)	35.18	17.59	311	0.035	12.95
MFMNABs (After adsorption)	35.66	17.83	311	0.013	20.68

FWHM * Full width at half maximum height.

**Table 2:** Comparison of d-spacing and particle size (D) obtained from HR-TEM and XRD.

HR-TEM		XRD	
**d-spacing (nm)**	**D (nm)**	**d-spacing (nm)**	**D (nm)**
0.242	12.68	0.254	12.95

**Table 3:** Adsorption isotherm parameters for As(III) adsorption by MFMNABs.

Isotherm	Values of Parameters			
Langmuir	q_max_ (mg g^−1^) 6.6533	K_L_ 0.0975	R^2^ 0.989	R_L_ 0.3389
Freundlich	K_F_ (mg g^−1^) (mg L^−1^)^n^ 0.7919	n 1.1507	R^2^ 0.9682	-
Temkin	B_1_ 0.4625	K_T_ (L mg^−1^) 0.3984	R^2^ 0.9711	-

**Table 4:** Kinetic parameters for adsorption of As(III) onto MFMNABs.

Models	Kinetics Parameters		
Pseudo-First-Order	*k*_1_ (min^−^^1^)	*q_e_* (mg g^−^^1^)	R^2^
	0.0223	2.492	0.9317
Pseudo-Second- Order	*k*_2_ (g mg^−1^ min^−1^)	*q_e_* (mg g^−^^1^)	R^2^
	0.08	0.1177	0.9998
Intra-particle Diffusion	*k_d_* (mg g^−1^ min^−1^)	C (mg g^−1^)	R^2^
	8.0456	0.1099	0.9758
Elovich model	A (mg g^−1^ min^−2^)	β (g mg^−1^ min^−1^)	R^2^
	1.01035	0.397	0.9601

**Table 5:** Comparison of the adsorption capacity of different adsorbents.

Adsorbents	Adsorption Capacity (mg/g)	References
Guava leaf biomass	1.05	[83]
Mango bark	1.25	[83]
Bagasse	1.35	[83]
Ferric hydroxide microcapsule-loaded alginate beads (FHMCA)	3.80	[84]
Modified saxaul ash	4.20	[85]
WTRs (water treatment residuals) loaded alginate beads	3.40	[86]
Iron impregnated AC from Lapsi seed stone	2.00	[87]
Magnetic nanoparticle obtained from metallic wool	2.20	[88]
Magnetite-maghemite nanoparticle	3.69	[89]
Hybrid (polymeric/inorganic) fibrous sorbent	75.67	[90]
Hybrid material zirconium polyacrylamide (ZrPACM-43)	41.48	[91]
Laterite soil (batch adsorption and fixed bed column)	0.18 69.22	[92]
Methionine functionalized magnetic nanoparticles	6.65	Present study

## Data Availability

Data will be provided upon request.
